# Background as Predictors of Food Safety Behavior in Peruvian University Students: The Mediating Role of Food Safety Attitudes

**DOI:** 10.3390/foods15040683

**Published:** 2026-02-13

**Authors:** Jairo I. González-Linares, Hypatia Ynfante-Acuña, Saúl Vara-Allhuirca, David Quispe-Sanca, Wilter C. Morales-García

**Affiliations:** 1 Escuela de Posgrado, Universidad Peruana Unión, Lima 15000, Peru; 2 Dirección General de Investigación, Universidad Peruana Unión, Lima 15020, Peru

**Keywords:** food safety behavior, food safety attitudes, food handling practices, university students, Peru

## Abstract

**Background:** Foodborne diseases (FBDs) pose a significant threat to public health and are particularly relevant among university students, who often face limitations in time, resources, and experience in food handling. This study examined the influence of background factors (experience and education) on food safety behavior and the mediating role of attitudes among Peruvian students. **Methods:** A cross-sectional study was conducted with 814 university students (53.8% female; 82.1% aged 17–22 years; 77.4% from private universities). Three validated scales were applied: background factors, attitudes, and behavior, all demonstrating acceptable reliability (α ≥ 0.71). Analyses were performed using PLS-SEM with 5000 bootstraps, confirming the convergent and discriminant validity of the constructs. **Results:** Results showed that background factors accounted for a small proportion (5.7%) of the variance in attitudes, and together with attitudes, they explained 31.8% of food safety behavior. The direct effects were significant: background → behavior (β = 0.266, *p* < 0.001), attitudes → behavior (β = 0.457, *p* < 0.001), and background → attitudes (β = 0.249, *p* < 0.001). Attitudes partially and modestly mediated the relationship, accounting for approximately 30% of the total effect of background factors on behavior (β_indirect = 0.114, *p* < 0.001). Women scored higher in personal hygiene, food hygiene, and cross-contamination prevention (*p* < 0.05), although multi-group comparisons revealed no significant differences in structural paths by gender. **Conclusions:** In conclusion, background factors are associated with food safety practices, and attitudes contribute to translating prior experience and education into behavior as a statistically significant but modest mediator, suggesting that other unmeasured factors (e.g., norms, habits, contextual constraints) also play an important role. Educational interventions should therefore prioritize strengthening attitudes and self-efficacy while also providing contextual resources that support safe practices in student kitchens.

## 1. Introduction

Foodborne diseases (FBDs) remain a preventable yet persistent threat to public health and human capital, with approximately 600 million cases and 420,000 deaths in 2010, representing 33 million DALYs, largely attributable to norovirus and *Campylobacter* spp. [1]. While public attention often focuses on food services and supply chains, a substantial proportion of the risk originates within households, where daily decisions about food selection, storage, preparation, and serving determine actual exposure to pathogens [2,3]. This household perspective is especially critical for university students, who transition from family or institutional meals to shared kitchens with limited time, budgets, and equipment, in environments strongly influenced by peers and social norms [4,5]. The literature consistently reveals gaps in knowledge, suboptimal attitudes, and risky practices among university students, particularly in areas such as temperature control, prevention of cross-contamination, and hand hygiene, with variations by region and academic discipline [6,7,8,9]. Young adults are less likely to verify cooking with a thermometer, tend to underestimate the risks associated with undercooked ground meat and eggs, and frequently fail to avoid cross-contamination, widening the gap between “knowing” and “doing” [3,10,11,12]. These gaps are not merely cognitive; they reflect systems of beliefs, perceived control, and social norms that shape the execution of safe behaviors under time pressure and limited resources [4,5].

The Theory of Planned Behavior (TPB) provides a solid framework to explain how attitudes, subjective norms, and perceived behavioral control shape intentions and actual behavior in the kitchen [13,14]. A systematic review and meta-analysis on food safety demonstrated that TPB constructs significantly predict intentions, with subjective norms playing a particularly strong role in behaviors such as using thermometers, separating raw from ready-to-eat foods, or adhering to the “5 °C rule” for refrigeration [15]. Extensions of TPB that incorporate explicit knowledge and affective risk appraisal reveal that beliefs about outcomes and emotions such as disgust or concern influence the persistence of inadequate household practices [16]. Among university students who are highly exposed to the influence of peers and digital networks, these social and perceived-control pathways become particularly powerful, either enhancing or inhibiting the translation of attitudes into behavior [15]. From this standpoint, variables such as formal education, previous experiences of foodborne disease (FBD), and cooking frequency are considered “distal antecedents” that do not directly determine behavior but rather shape the beliefs and perceptions that feed into attitudes and perceived control [13,14].

Background factors, such as formal education and practical experience, operate as distal determinants shaping the beliefs and self-efficacy that sustain attitudes [13,14]. Classical studies show that students in food-related disciplines (e.g., nutrition, food science, hospitality) outperform their peers from other fields in knowledge and, sometimes, in practice, likely due to curricular exposure and vocational self-selection [9,17]. Recent evidence replicates this pattern and highlights the effect of cumulative experiential learning: higher frequency of cooking and previous training are associated with greater knowledge and, in some cases, safer practices, although the translation into consistent behavior is never automatic [5,18]. The persistent “knowledge practice gap” reflects how contextual constraints, competing motivations, and weak norms prevent knowledge from materializing into action [12,18]. In this context, the different components that make up the antecedents—formal courses, sources of knowledge, cooking frequency, and previous experiences with FBD—can be understood theoretically as manifestations of a single “experiential capital” or prior background in food safety that predisposes individuals to certain beliefs, attitudes, and levels of self-efficacy [4,5,10,11]. In line with the TPB and its extensions, in which several related indicators are grouped into common distal factors, these antecedents are modeled in the present study as a single reflective latent construct of food safety background that summarizes students’ prior preparation in this domain [7,9,19].

The informational dimension alone has shown diminishing returns. Meta-analyses of educational interventions, particularly online ones, report modest improvements in knowledge and small or inconsistent effects on attitudes and practices. This suggests that information transfer needs to be complemented by motivational and environmental levers [20,21]. Among university students, mobile interventions improved KAP (Knowledge, Attitudes, and Practices) components but revealed the fragility of behavior change when contextual barriers and social reinforcers are not addressed [22]. These findings support multicomponent strategies: skill training (e.g., thermometer use, effective disinfection), environmental restructuring (e.g., access to supplies and equipment in residences), and normative messaging leveraging peer influence all aligned with the TPB framework [15]. Internal heterogeneity within the student population adds complexity. International students and first-year students face unfamiliar foods, rules, and equipment. Those experiencing financial insecurity or heavy workloads tend to prioritize cost and convenience, while certain cultural practices, such as consuming raw or undercooked foods, increase risk if not paired with appropriate controls, reshaping attitudes and perceived control [3,12]. Differences by gender and maternal education have been linked to better practices and greater receptivity to training, suggesting opportunities to tailor content and support [23,24,25]. The COVID-19 pandemic, by increasing home cooking and attention to hygiene, demonstrated the malleability of attitudes under heightened perceived risk and, at the same time, the resilience of habits and structural constraints, as well as the ambivalent role of digital ecosystems in shaping norms and perceived control [8].

Historically, the KAP (Knowledge, Attitudes, Practices) model has been used to diagnose gaps and guide interventions. However, its linear assumptions underestimate the motivational and environmental complexity of everyday behavior, leading to the increasing adoption of TPB and the Theory of Reasoned Action, which integrate self-efficacy, norms, and perceived control [20,26,27,28]. university students, differences in discipline, exposure to courses, and cooking experience serve as “ground setters” that shape beliefs and intentions, explaining why students with similar knowledge levels may diverge in practical execution [5,9]. The contemporary emphasis has shifted from merely “increasing information” to “activating motivations, capacities, and favorable norms” to close the intention–action gap in real-world contexts [15,21]. In this study, attitudes toward food safety are conceptualized as cognitive (perceived importance, outcome beliefs) and affective (concern, disgust) components linked to safe food handling [16]. Background factors are defined as formal food safety education (courses, workshops), field of study (food-related vs. non-food-related), cooking frequency, and self-reported history of FBDs, functioning as distal determinants that influence beliefs and self-efficacy [9]. Behavior is operationalized through practices with proven links to risk reduction: hand hygiene, prevention of cross-contamination, temperature control, and safe cooling/reheating practices where most failures are observed among university students [4,7,10]. In sum, the challenge is not only to transmit “what to do,” but to foster conditions under which “doing the right thing” is feasible and socially approved in student kitchens, aligning motivations, capabilities, and norms with what the evidence identifies as behaviors with the greatest preventive impact [15,20]. From this perspective, attitudes constitute one of the proximal mechanisms through which education and experience can be translated—only partially—into safe practices, while other unmeasured determinants, such as social norms, habits, and environmental constraints, also contribute substantially to behavior [20,21]. Consequently, our study assumes from the outset that the mediating effect of attitudes will likely be moderate in magnitude and will coexist with direct pathways between antecedents and behavior that do not operate through attitudes.

This orientation places attitudes as a proximal lever for change, nourished by education and experience, and suggests that closing the knowledge–practice gap requires intervening on beliefs and contexts, not only on content [20,21]. This orientation places attitudes as a proximal lever for change, nourished by education and experience, and suggests that closing the knowledge–practice gap requires intervening on beliefs and contexts, not only on content [15,16].

**Figure 1:**
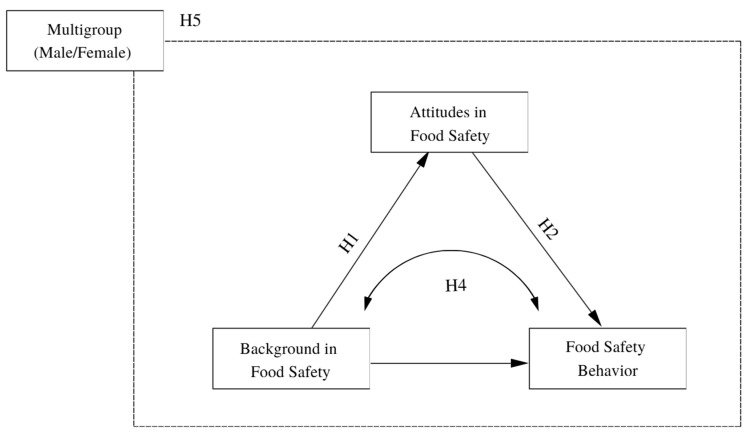
Conceptual Model.

Based on this literature review, we examined the following research hypotheses (Figure 1):

**H1.**  *Food safety background factors are positively associated with food safety behavior among Peruvian university students.*

**H2.**  *Food safety attitudes are positively associated with food safety behavior among Peruvian university students.*

**H3.**  *Food safety background factors are positively associated with food safety attitudes among Peruvian university students.*

**H4.**  *Attitudes mediate the relationship between background factors and food safety behavior.*

**H5.**  *Gender moderates the relationship between attitudes, background factors, and food safety behavior.*

## 2. Methods

### 2.1. Participants

An explanatory and cross-sectional study was conducted with the objective of analyzing the relationships between background factors, attitudes, and food safety behavior among Peruvian university students. This design also allows for the evaluation of possible mediation effects through a structural equation modeling (SEM) approach [29]. Participants were selected using a non-probabilistic sampling method. The minimum sample size was calculated using Soper’s electronic tool [30], taking into account the number of observed and latent variables in the SEM model, the expected effect size (λ = 0.30), the statistical significance level (α = 0.05), and the statistical power (1 − β = 0.80). The result indicated a minimum of 119 participants. The majority of participants were female (53.8%). Regarding age, the predominant group was between 17 and 22 years old (M = 20.60; SD = 3.044). In terms of geographic origin, most participants came from the Sierra region (43.2%). Finally, most students were enrolled in private universities (77.4%) (Table 1).

### 2.2. Procedure

Data collection was carried out between August and November 2024 using a Google Forms questionnaire. The invitation to participate was distributed through Facebook, institutional emails, and university announcements. Before initiating the application process, permissions were requested and obtained from the administrations of the participating universities, ensuring formal authorization and access to conduct fieldwork. A total of 15 questionnaires were excluded due to being incomplete or containing inconsistent responses, thus ensuring the quality of the data analyzed. The study was approved by the Ethics Committee of Universidad Peruana Unión under Resolution No. 748-2024/UPeU-EPG-CEPG-D. All students signed a digital informed consent form before participating, and confidentiality and anonymity of their information were guaranteed. The study was conducted following the ethical principles of the Declaration of Helsinki.

### 2.3. Measures

The adaptation of the instruments was carried out following international methodological guidelines for the translation and cultural adaptation of scales [31].

1. Two bilingual translators, native Spanish speakers, independently translated the original instruments into Spanish. Both versions were then compared, and a consensus version was developed to ensure semantic and conceptual accuracy.
2. The consensus Spanish version was back-translated into English by two native English speakers proficient in Spanish who had no prior knowledge of the instruments. This step aimed to verify that the original meaning of the items remained unchanged.
3. A committee composed of two specialists and the study authors evaluated both the Spanish version and the back-translations. The committee reviewed content, clarity, and cultural appropriateness, producing a preliminary version ready for piloting.
4. The preliminary version was administered to a pilot group of 5 university students with characteristics similar to those of the final sample. No ambiguous terms or comprehension difficulties were identified during this process, indicating that the items were appropriate for the Peruvian university population. Therefore, no further modifications were made. As a result, final versions of the three instruments were obtained, suitable for use in the Peruvian context. These are described below, and the complete model is provided in Appendix A.

Background in Food Safety (BFS): The Food Safety Background Factors Scale adapted from Marklinder et al. [32] was used. It consisted of eight items distributed across three dimensions: cooking and handling experience, courses taken on food safety, and sources of food safety knowledge. The items were rated on a 5-point Likert scale: the first four items from 1 = Never to 5 = Every day, and the remaining four from 1 = Strongly disagree to 5 = Strongly agree. In the original studies, these indicators are integrated into a single latent “Background” factor that combines practical experience, educational exposure, and main sources of information, showing that these prior exposures form a common underlying domain that predicts food safety knowledge, attitudes, and behavior [32]. In line with this conceptualization, in the present study, these items were modeled as reflective indicators of a single latent construct of background or “experiential capital” in food safety, summarizing students’ overall level of prior exposure to food safety content and practices. This decision is consistent with recommendations for specifying higher-order constructs in PLS-SEM when several related facets represent the same conceptual domain [32,33,34]. The internal consistency and validity of this construct were evaluated using internal reliability indices and convergent and discriminant validity, with results presented in the Results section.

Food Safety Attitudes (AFS): The Food Safety Attitudes Scale adapted from Sanlier and Baser [35] was used. It comprises 11 items rated on a 5-point Likert scale (1 = Strongly disagree to 5 = Strongly agree). This reflective scale was designed to assess participants’ perceptions and attitudes regarding food safety. In this study, the internal reliability of the scale was evaluated using Cronbach’s alpha and composite reliability (ρc), and convergent validity was assessed through the Average Variance Extracted (AVE). Values of ρc ≥ 0.70 and AVE ≥ 0.50 were considered acceptable, in accordance with recommendations for PLS-SEM models [33]. The specific values of these indices are reported in the Results section.

Food Safety Behavior (FSB): Behavior was assessed using the Food Safety Behavior Scale, which measured participants’ practices related to food safety. This reflective instrument consisted of 21 items distributed across 5 dimensions: personal hygiene, equipment hygiene, food hygiene, cross-contamination, and storage conditions. Items were rated on a 5-point Likert scale (1 = Strongly disagree to 5 = Strongly agree). As with the attitudes scale, internal reliability was evaluated using Cronbach’s alpha and composite reliability (ρc), and convergent validity was assessed via AVE. Since in PLS-SEM ρc is considered a more appropriate indicator than Cronbach’s alpha for scales with few items per dimension, greater weight was given to the combination of ρc (≥0.70) and AVE (≥0.50) when assessing the internal consistency of the subscales [35]. In cases where α values slightly below the conventional threshold were observed in any subdimension, the decision to retain items was based on the adequacy of ρc and AVE, as well as the importance of preserving content coverage; the results of these analyses are detailed in the Results section.

### 2.4. Statistical Analysis

The reflective nature of the constructs was confirmed through a confirmatory tetrad analysis (CTA-PLS), in which less than 80% of the tetrads were significant (*p* < 0.05), a criterion that supported retaining the reflective specification of the background, attitudes, and behavior constructs [36]. Mardia’s test showed a lack of multivariate normality; therefore, descriptive-comparative analyses were conducted using the median as the measure of central tendency. The homogeneity of responses between men and women was assessed using Mood’s median test (α = 0.05).

Subsequently, the measurement model and the structural model were evaluated using structural equation modeling with the partial least squares technique (PLS-SEM), employing the SEMinR package in the R programming language (version 4.5.2; R Foundation for Statistical Computing, Vienna, Austria) and the open-source RStudio environment (version 2025.09.2) [37]. A two-step approach was followed: first, the measurement model was evaluated and, once its adequacy was established, the structural model was estimated [36].

In the measurement model, factor loadings were calculated for individual items, using a recommended reference value of 0.70. Convergent validity was assessed through the Average Variance Extracted (AVE), and discriminant validity through the Heterotrait–Monotrait Ratio (HTMT). AVE values ≥ 0.50 and HTMT values below 0.85–0.90 were considered acceptable, in accordance with PLS-SEM guidelines [36]. With respect to reliability, Cronbach’s alpha and composite reliability (ρc) were used in a complementary manner, adopting ρc (≥0.70) together with AVE (≥0.50) as the main criteria for interpretation, since in PLS-SEM ρc more accurately captures the reliability of reflective constructs with a reduced number of items.

In the structural model, path coefficients were estimated among background, attitudes, and behavior, as well as coefficients of determination (R^2^) to assess the proportion of explained variance in the endogenous variables. Effect sizes (f^2^) of each predictor on the endogenous constructs were also calculated, using conventional cut-off points for small, medium, and large effects. Mediation hypotheses were analyzed using a systematic bootstrapping procedure with 5000 resamples, obtaining direct, indirect, and total effects along with their 95% confidence intervals. This procedure made it possible to estimate the partial mediation of attitudes in the relationship between background and behavior, as well as to quantify the magnitude of the mediated effect.

Finally, multigroup analyses were conducted in PLS-SEM to explore potential gender moderation, estimating the model separately for men and women and comparing path coefficients between groups via bootstrapping. Differences in structural paths were evaluated based on confidence intervals and *p*-values associated with the coefficient differences, which allowed determining whether gender did or did not modify the strength of the associations among background, attitudes, and behavior.

**Figure 2:**
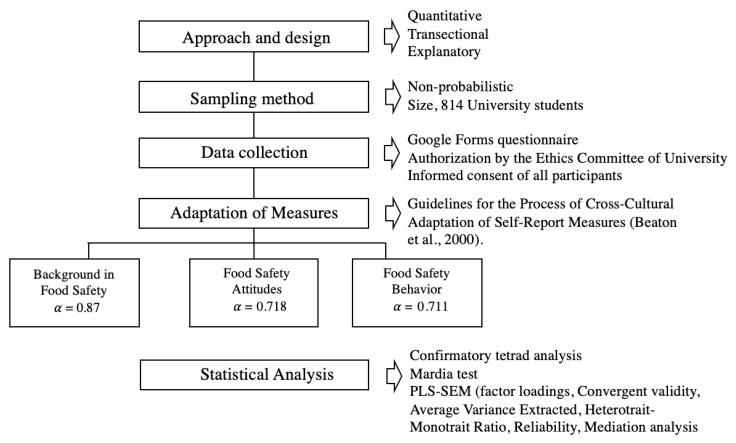
Workflow [31].

The study was conducted following the workflow shown in Figure 2.

## 3. Results

### 3.1. Descriptive-Comparative Analysis of the Study Variables

Table 2 presents the distribution of scores for the study variables and their respective dimensions. Overall, participants show an intermediate level of food safety background (Median = 3.38). Only the cooking experience (CE) dimension showed significant sex differences (*p* < 0.05), indicating that women reported performing food handling tasks more frequently. In the food safety attitudes scale, a median of 4 was observed in both sexes, with an IQR ≤ 1, which reflects favorable attitudes and low variability, with no significant differences between men and women. Regarding food safety behavior, scores fell between “almost always” and “always,” with particularly high values in the dimensions of personal hygiene (PH) (Median = 4.25) and food hygiene (FH) (Median = 4.33). Women also scored significantly higher (*p* < 0.05) in PH, FH, and cross-contamination (CC), indicating a higher self-reported frequency of these practices compared with men, without implying qualitative superiority in food safety skills.

### 3.2. Evaluation of the Measurement Model

Table 3 presents the standardized factor loadings (λ), internal reliability coefficients (Cronbach’s alpha and composite reliability, CR), and the average variance extracted (AVE) for each construct and its subdimensions, reported by sex and for the overall sample. Interpretation of these results was based on commonly accepted cut-off values in the literature: λ ≥ 0.70, α ≥ 0.70, CR ≥ 0.70, and AVE ≥ 0.50 [33]. The items adapted and used in Spanish showed significant overall Aiken’s content validity coefficients for Background (V ≥ 0.85, *p* < 0.05), Attitudes (V ≥ 0.94, *p* < 0.05), and Behavior (V ≥ 0.96, *p* < 0.05), confirming that the experts considered the items to be pertinent and representative of the constructs assessed. Most items displayed high and significant factor loadings (λ ≥ 0.70, *p* < 0.01). The lowest values (λ ≈ 0.63–0.69) were observed for AFS6, AFS7, AFS9, AFS10, FSB5, FSB8, FSB13, and FSB15; however, since they exceeded the 0.60 threshold and contributed to the conceptual coherence of the dimensions, they were retained in the model.

With respect to construct reliability, some subdimensions (e.g., CE, CC and specific attitude items) showed Cronbach’s alpha values slightly below the 0.70 threshold reported in the literature [33]. This pattern is consistent with the well-known sensitivity of α to the number of items, which tends to underestimate reliability in short subscales [38]. Importantly, in all cases the composite reliability (CR) exceeded the recommended minimum value and the AVE was above 0.50, supporting adequate internal consistency and convergent validity for these subdimensions [33,38]. In line with current recommendations for PLS-SEM, where CR is considered a more appropriate indicator of reliability than α for reflectively measured constructs with few items, we adopted CR and AVE as the primary criteria to judge the quality of the measurement model [39]. As an additional check, sensitivity analyses excluding items with lower loadings did not materially change the structural relationships or improve reliability indices enough to justify item removal; therefore, all items were retained to preserve content coverage.

Table 4 and Table 5 complement this evaluation through an analysis of discriminant validity. Cross-loadings show that each item loads more strongly on its theoretical construct than on the others, while the Fornell–Larcker criteria (square roots of the AVE greater than the correlations between constructs) and the HTMT values remain within the recommended ranges. Taken together, these results support the convergent and discriminant validity of the measurement model for both sexes and for the total sample.

### 3.3. Evaluation of the Structural Model

**Figure 3:**
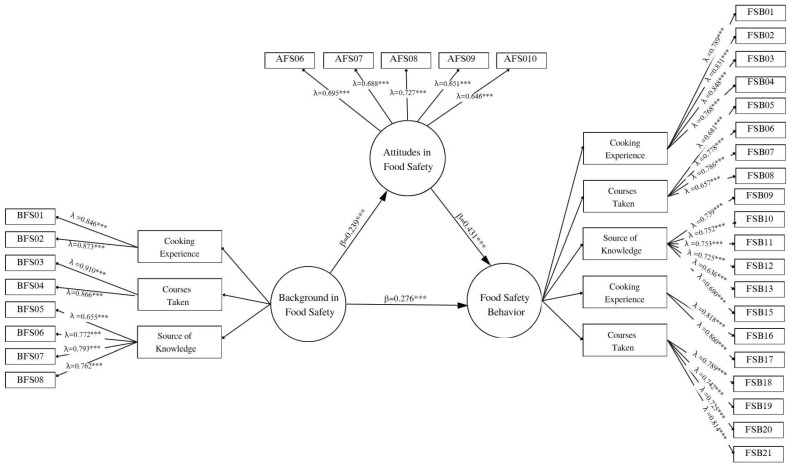
Structural Model with Bootstrapping. Note. Λ = standardized factor loadings; β = standardized path coefficients. *** *p* < 0.001.

Figure 3 shows the path diagram of the structural equation model estimated using partial least squares (PLS-SEM). The model represents the structural relationships among the constructs Background, Attitudes, and Behavior in food safety, including their respective dimensions. The results indicate that Background explains 5.7% of the variance in Attitudes (R^2^ = 0.057). This value indicates that food safety background (capital experiencial) has a statistically significant but modest capacity to predict attitudes, leaving a substantial proportion of their variance to other unmeasured factors. In turn, the combination of Background and Attitudes increases the explained variance of Food Safety Behavior to 31.8%, which reflects a moderate level of explained variance for behavior in everyday food handling.

Table 6 summarizes the estimated direct effects (H1–H3). In both sexes, Background exerts a positive, moderate, and statistically significant effect on Behavior (β > 0.260, T > 6.5, *p* < 0.001), while Attitudes show an even stronger effect. Although the standardized coefficient for Attitudes is larger than that of Background, both predictors contribute meaningfully to explaining Food Safety Behavior and should be interpreted as complementary rather than as a single dominant mechanism. The coefficients were slightly higher in women (e.g., AFS → FSB: β = 0.472, T = 11.420, *p* < 0.001), but these differences were not statistically significant in the multigroup comparisons (see Table 8), indicating thatthe pattern of associations among Background, Attitudes, and Behavior is structurally similar across sexes.

### 3.4. Mediation Analysis

Table 7 shows that the BFS → AFS → FSB pathway is significant in men (β = 0.141, T = 5.293, *p* < 0.001), in women (β = 0.100, T = 3.181, *p* < 0.001), and in the overall sample (β = 0.114, T = 5.692, *p* < 0.001).

Since the direct effect of BFS on FSB (H1) was also significant, these results indicate the presence of partial mediation: background influences behavior both directly and indirectly through attitudes. The indirect pathway BFS → AFS → FSB accounts for approximately 30% of the total effect of background on behavior (0.114 ÷ 0.38 ≈ 0.30) in the overall sample, which corresponds to a statistically robust but modest mediation effect. In other words, attitudes help to translate prior food safety experience and education into safer practices, while a considerable proportion of the influence of background on behavior operates through other mechanisms not captured in this model.

### 3.5. Multigroup Analysis

Table 8 presents the results of the multigroup analysis used to test gender moderation (Hypothesis 5). No statistically significant differences at the 5% level were observed between men and women in any of the paths evaluated. Although the difference in the coefficient for the BFS → AFS path approaches the 10% significance threshold (path diff. = 0.106; *p* = 0.088), the evidence is not sufficient to support that the dynamics of attitude formation, or their translation into behaviors, differ consistently between men and women in this sample of Peruvian university students. Taken together with the descriptive results, this suggests that, although women report higher average levels in some behavioral dimensions (e.g., hygiene and cross-contamination), the underlying structural relationships among background, attitudes and behavior are comparable across sexes.

## 4. Discussion

The results confirm Hypothesis 1, showing that food safety background is positively associated with food safety behavior among Peruvian university students. In our model, higher levels of background, understood as a combination of cooking experience, prior exposure to foodborne disease, and formal or informal education about food safety, are related to more frequent self-reported safe practices. However, the size of this association is moderate, and the explained variance in food safety behavior remains partial, indicating that background is a helpful but not sufficient condition for consistent safe food handling. This pattern is compatible with the “knowledge-practice gap” described in the literature, whereby exposure to food safety content and accumulated experience improves what students know but does not automatically ensure that safe practices are applied under real-life constraints [4,5]. In our data, the coexistence of intermediate levels of background with persistent risky behaviors (such as limited thermometer use or incomplete prevention of cross-contamination) suggests that prior experience may sometimes consolidate shortcuts or permissive norms when it is not accompanied by structured training and feedback [8]. This is consistent with studies that emphasize the ambivalent role of informal learning and digital sources: they can provide useful information but also normalize unsafe practices or minimize perceived risk [15,22]. From the perspective of the Theory of Planned Behavior, one plausible interpretation is that background contributes to shaping beliefs and perceived control, but its impact on behavior is filtered by subjective norms, convenience, and competing goals (e.g., time pressure, taste, and cost) [16]. In practical terms, these findings support the idea that interventions should not rely solely on increasing exposure or general experience, but rather on integrating hands-on training, clear feedback on performance, and environmental supports that make safe options easier to implement in student kitchens.

Hypothesis 2 is also confirmed, showing that attitudes toward food safety are positively associated with food safety behavior among Peruvian university students. In line with studies conducted in different regions, more favorable attitudes are linked to better performance in temperature control, hand hygiene, and prevention of cross-contamination [4,8,9,10]. In our model, attitudes show a stronger standardized coefficient than background, but both predictors contribute jointly to explaining food safety behavior, which reaches an R^2^ of 0.318. This level of explained variance is moderate and indicates that attitudes are an important proximal correlate of behavior rather than a sole determinant. The pattern observed is compatible with the idea that students who attribute higher importance to food safety, perceive greater benefits in preventive behaviors, and experience more concern about foodborne disease are more likely to report safer practices, especially when they also feel capable of performing them. At the same time, the persistence of unsafe behaviors despite generally favorable attitudes suggests that other factors—such as habits, perceived effort, and social expectations in shared kitchens—continue to play a substantial role [5,11]. Overall, our findings reinforce calls for interventions that explicitly target attitudes and perceived control, not just knowledge, by working on risk appraisal, emotional engagement, and the practical feasibility of safe behaviors in everyday student routines.

Hypothesis 3 is confirmed by showing that food safety background is positively associated with attitudes toward food safety among Peruvian university students. This result is consistent with international evidence indicating that academic training and accumulated practice tend to foster more positive attitudes toward safe food handling [5,9,17], At the same time, the magnitude of this association is modest: in our model, background explains only 5.7% of the variance in attitudes (R^2^ = 0.057), which means that most of the variability in attitudes is attributable to other determinants not captured here. This is coherent with theoretical models that highlight the contribution of social norms, self-efficacy, perceived behavioral control, and past habits in shaping attitudes and intentions [15,16]. Lived experiences such as having suffered a foodborne disease may activate affective components (e.g., disgust, concern), whereas formal education provides procedural frameworks that can strengthen positive evaluations of safe practices. Nevertheless, our findings suggest that—at least in this sample—background operates as a modest predictor among many others and that changing attitudes will likely require acting simultaneously on social influences, emotional responses, and structural barriers, rather than relying exclusively on increasing exposure or training hours.

Hypothesis 4 is also supported, showing that attitudes mediate the relationship between background (experience and education) and food safety behavior. Because both the indirect (mediated) effect and the direct effect of background on behavior were statistically significant, the mediation observed corresponds to complementary partial mediation [40]. In practical terms, this means that background exerts its influence on behavior through two routes: a direct pathway and an indirect pathway via attitudes. The indirect path BFS → AFS → FSB explains approximately 30% of the total effect of background on behavior in the overall sample, which indicates that the mediation is statistically robust but of modest magnitude. Thus, attitudes help to translate prior experience and educational exposure into safer practices, but they account for only a portion of this translation. A considerable part of the effect of background on behavior is likely transmitted through other mechanisms not explicitly modeled here, such as social norms, habits, peer behavior, perceived time constraints, and self-efficacy for implementing specific practices [5,15,41]. These results are congruent with studies showing that students with training or experience in food handling may still maintain unsafe behaviors when the perceived benefits of change are low, safe options are inconvenient, or their social environment does not reinforce prevention [4,6,7]. From an intervention standpoint, our findings suggest that strengthening attitudes is a relevant—but not sufficient—lever for improving food safety behavior: programs will need to combine attitudinal work (reframing beliefs and perceived value of prevention) with practical training, peer-based normative reinforcement, and environmental changes that reduce friction and make safe practices more attainable in daily life.

Regarding Hypothesis 5, although level differences were observed between men and women in some dimensions of food safety behavior (e.g., higher scores in personal hygiene, food hygiene, and cross-contamination among women), gender did not moderate the structural relationships among background, attitudes, and behavior. That is, the pattern of associations between the constructs was statistically similar in both groups, and multigroup comparisons did not reveal significant differences in the main paths at the 5% level. This is consistent with studies in university populations and young adults that report gender differences in average levels of knowledge or self-reported practices, but not systematic moderation of the links proposed by the Theory of Planned Behavior [5,15,41]. A plausible interpretation is that women’s higher scores in hygiene-related behaviors reflect gendered socialization patterns around cleanliness and food preparation, as well as a greater involvement in cooking tasks, whereas the psychological mechanisms that connect background, attitudes, and behavior (e.g., perceived norms, perceived control) operate in a comparable way in men and women under similar living conditions. In our context, shared constraints such as time pressure, limited equipment, and crowded refrigerators may shape the weight of attitudes and perceived control in similar directions for both sexes, thereby attenuating potential moderating effects of gender. From a practical perspective, these results suggest that educational interventions can be designed with broadly similar core components for male and female students, while taking into account that women may start from slightly higher baseline levels of certain safe behaviors. It is important to avoid interpreting these mean differences as evidence of functional or behavioral superiority of any gender; rather, they point to different starting points that do not translate into distinct structural pathways in the model. Future research in other cultural and socioeconomic settings may help to clarify under which conditions gender becomes a more salient moderator of domestic food safety behavior.

### 4.1. Implications

The findings of this study offer a set of implications that can be organized across three interdependent levels: professional practice, institutional and sectoral policy, and theory.

First, in terms of professional practice, the results show that simply transmitting knowledge does not guarantee the adoption of safe behaviors; therefore, training programs should not focus exclusively on information delivery but place greater emphasis on strengthening attitudes as an important proximal driver of change, while also addressing skills and contextual barriers. This implies developing active teaching strategies that integrate procedural training (use of thermometers, prevention of cross-contamination, effective hygiene), emotionally engaging experiences that reinforce the perceived relevance of food safety, and supervised practice spaces that increase instrumental self-efficacy. In turn, professional practice should extend into everyday support through contextual reminders, peer tutoring, and the creation of environments that make it easier and more accessible to “do the right thing” under time pressure and resource constraints.

Second, at the level of university and sectoral policy, the results support the need to institutionalize minimum guidelines that ensure basic structural conditions for safe behavior: student kitchens equipped with adequate refrigerators, availability of functioning thermometers, color-coded surfaces, and auditable cleaning protocols. Policy should go beyond isolated informational campaigns and incorporate mechanisms for ongoing monitoring and feedback, as well as incentives for student participation in food safety initiatives. Universities could integrate food safety modules into general education courses, distribute low-cost kits for first-year students, and establish student ambassador programs that model safe norms and behaviors. From a sectoral perspective, authorities could promote the development of guidelines tailored specifically to university populations, ensure their inclusion in institutional accreditation processes, and prioritize funding for evidence-based interventions that activate attitudes and reduce contextual barriers.

Third, the theoretical implications point to the consolidation and expansion of explanatory models of food safety behavior. The evidence supports the Theory of Planned Behavior but also highlights the need to enrich it with constructs related to social norms, perceived control, and habit formation, given that background factors explain only a limited proportion of the variance in attitudes and behaviors. This reinforces the view that translating knowledge into practice depends on motivational, social, and environmental factors, inviting the development of multilevel conceptual frameworks that articulate individual variables with contextual determinants. The identification of a statistically significant yet modest partial mediation also suggests the existence of additional, unmodeled pathways—such as the influence of emotions (e.g., disgust, concern), peer normative pressure, and practical constraints—that should be incorporated into future theoretical refinements. Moreover, the absence of gender moderation in the structural paths indicates that the basic psychological mechanisms linking background, attitudes, and behavior may operate similarly in men and women living under comparable conditions, which has implications for designing broadly applicable, gender-inclusive theoretical models.

### 4.2. Results Limitations

This study presents several limitations that should be considered when interpreting the findings. First, the cross-sectional design impedes causal interpretation of the observed relationships, so the mediation of attitudes can only be understood at a statistical level. Future studies should employ longitudinal or experimental designs to assess directionality and changes over time. Second, the use of non-probabilistic convenience sampling and online recruitment may limit representativeness, with an overrepresentation of women, younger students, and those from private universities. This constrains the generalization of the results to all Peruvian university students, and future research should use probabilistic or stratified sampling strategies that include a greater diversity of institutions and regions.

Third, the use of self-report measures introduces risks of social desirability bias and common method variance, which may have inflated some of the observed associations. For future studies, incorporating objective and observational methods (e.g., refrigerator audits, practical cooking tasks, electronic temperature logs) would help strengthen the validity of the measures. Fourth, although composite reliability and AVE values met recommended thresholds, some subdimensions presented Cronbach’s alpha values slightly below 0.70 and heterogeneous response formats, which may have attenuated certain relationships and indicate the need for further refinement of the instruments. It would be advisable to standardize response scales, re-examine items with lower internal consistency, and explore alternative specifications—such as formative or composite models—for background variables, given their compositional nature.

Fifth, the explained variance was modest for attitudes and moderate for behavior, suggesting the influence of relevant variables not included in the model (such as peer norms, socioeconomic conditions, habit strength, or availability of equipment). Incorporating these factors in future models would improve the precision of the estimates and provide a more comprehensive picture of domestic food safety behavior in young adults. Finally, the study is restricted to Peruvian university students, so generalization to other countries and cultural contexts should be made with caution. Multicenter replications and cross-cultural invariance analyses would be key steps to assess the robustness and transferability of the findings.

## 5. Conclusions

In summary, the results show that food safety background is associated with university students’ practices, but its impact is only partial and remains limited when considered in isolation. Attitudes toward food safety emerge as an important proximal factor associated with safer behaviors and operate as a statistically significant, yet modest, mediator of the relationship between background and behavior. This partial and moderate mediation indicates that attitudes help translate prior exposure and experience into practice, while a substantial proportion of the influence of background on behavior is likely transmitted through other mechanisms not captured in this model. These findings underscore the need to design interventions that do not focus solely on transmitting information, but instead promote strong beliefs, self-efficacy, and positive motivations, reinforced by environments and social norms that facilitate responsible behavior in student contexts.

The main contribution of this study lies in empirically documenting, in a Peruvian university population, that the relationships between background, attitudes, and food safety behavior are consistent with a model of partial mediation of modest magnitude and that these structural relationships remain stable across genders. This provides relevant evidence for the design of educational and public health strategies that can be applied to both male and female students, while recognizing differences in baseline levels of certain practices. Looking ahead, longitudinal and intervention studies are needed to assess whether changes in attitudes and self-efficacy lead to sustained improvements in food safety behavior, as well as research that explicitly incorporates other key variables from the Theory of Planned Behavior (subjective norms, perceived behavioral control) and contextual factors (living conditions, access to equipment, time constraints). It would also be pertinent to replicate and extend this model in other universities and regions of Latin America, to explore the potential of digital tools and experiential or simulation-based training programs, and to evaluate their impact both on reducing risky practices and on the incidence of foodborne diseases in young populations.

## Figures and Tables

**Table 1:** Sociodemographic Characteristics of the Participants.

Characteristics		n	%
Sex	Male	376	46.2
	Female	438	53.8
Age	17–22 years	668	82.1
	23–28 years	129	15.8
	>28 years	17	2.1
Region of origin	Coast	235	28.9
	Sierra	352	43.2
	Jungle	227	27.9
Type of institution	Private	630	77.4
	Public	184	22.6

**Table 2:** Descriptive Analysis.

	Men		Women		Total Sample	
	Median	IQR	Median	IQR	Median	IQR
Background	3.38	0.88	3.38	0.89	3.38	0.88
CE	3.00 ^a^	0.50	3.50 ^b^	1.00	3.50 ^b^	1.00
CT	3.00	1.00	3.00	1.00	3.00	1.00
SK	3.50	1.00	3.50	1.00	3.50	1.00
Attitudes	4.00	1.00	4.00	0.86	4.00	0.86
Behavior	4.05	0.89	4.20	0.75	4.15	0.85
PH	4.00 ^a^	1.00	4.25 ^b^	1.00	4.25 ^b^	1.00
EH	4.00	1.00	4.00	1.00	4.00	1.00
FH	4.33 ^a^	0.83	4.42 ^b^	0.83	4.33 ^a^	0.83
CC	4.00 ^a^	1.50	4.50 ^b^	1.50	4.00 ^a^	1.50
SC	4.00	1.00	4.00	1.25	4.00	1.25

Note. IQR: Interquartile Range, CE: Cooking Experience; CT: Courses Taken; SK: Source of Knowledge; PH: Personal Hygiene; EH: Equipment Hygiene; FH: Food Hygiene; CC: Cross-Contamination; SC: Storage Conditions. Different superscript letters in the same row indicate a significant difference in medians using Mood’s median test at a 95% confidence interval.

**Table 3:** Factor loadings, reliability, and validity of the measurement model.

Construct/Item	Men				Women					Overall Sample			
	λ	α	CR	AVE	λ	α	CR	AVE	Aiken’s V	λ	α	CR	AVE
Background (BFS)		0.661	0.812	0.594		0.61	0.788	0.56	0.85		0.63	0.799	0.574
BFS1	0.807				0.879					0.846			
BFS2	0.888				0.852					0.873			
Cooking Experience (CE)	0.639	0.615	0.836	0.719	0.627	0.666	0.857	0.749		0.634	0.648	0.85	0.739
Courses Taken (CT)	0.79	0.779	0.898	0.815	0.686	0.691	0.866	0.763		0.739	0.734	0.882	0.789
BFS3	0.938				0.861					0.91			
BFS4	0.865				0.886					0.866			
Source of Knowledge (FC)	0.866	0.723	0.828	0.548	0.903	0.741	0.837	0.564		0.879	0.733	0.834	0.558
BFS5	0.647				0.659					0.655			
BFS6	0.762				0.777					0.772			
BFS7	0.786				0.797					0.793			
BFS8	0.759				0.764					0.762			
Attitudes (AFS)		0.762	0.839	0.511		0.753	0.835	0.505	0.94		0.756	0.837	0.507
AFS6	0.757				0.635					0.688			
AFS7	0.72				0.655					0.688			
AFS8	0.736				0.787					0.765			
AFS9	0.675				0.771					0.729			
AFS10	0.681				0.693					0.686			
Behavior (FSB)		0.885	0.915	0.684		0.864	0.902	0.647	0.96		0.874	0.909	0.665
Personal Hygiene (PH)	0.794	0.817	0.878	0.643	0.801	0.828	0.886	0.661		0.799	0.825	0.884	0.656
FSB1	0.743				0.82					0.789			
FSB2	0.841				0.828					0.831			
FSB3	0.815				0.871					0.848			
FSB4	0.803				0.727					0.768			
Equipment Hygiene (EH)	0.826	0.741	0.836	0.562	0.802	0.666	0.799	0.501		0.814	0.704	0.818	0.53
FSB5	0.665				0.7					0.681			
FSB6	0.786				0.768					0.778			
FSB7	0.825				0.751					0.786			
FSB8	0.713				0.6					0.657			
Food Hygiene (FH)	0.838	0.805	0.86	0.507	0.839	0.813	0.865	0.517		0.838	0.81	0.863	0.514
FSB9	0.709				0.763					0.739			
FSB10	0.744				0.76					0.752			
FSB11	0.744				0.756					0.753			
FSB12	0.73				0.719					0.725			
FSB13	0.625				0.636					0.636			
FSB15	0.713				0.672					0.69			
Cross-Contamination (CC)	0.844	0.626	0.842	0.727	0.806	0.529	0.809	0.679		0.827	0.582	0.827	0.704
FSB16	0.824				0.806					0.818			
FSB17	0.88				0.842					0.86			
Storage Conditions (SC)	0.831	0.778	0.856	0.598	0.773	0.764	0.849	0.585		0.8	0.77	0.852	0.59
FSB18	0.768				0.804					0.789			
FSB19	0.741				0.748					0.742			
FSB20	0.763				0.69					0.725			
FSB21	0.819				0.811					0.814			

Note. λ = factor loading; α = Cronbach’s alpha; CR = composite reliability; AVE = Average Variance Extracted; BFS = Background in Food Safety; AFS = Attitudes in Food Safety; FSB = Food Safety Behavior; PH = Personal Hygiene; EH = Equipment Hygiene; FH = Food Hygiene; CC = Cross-Contamination; SC = Storage Conditions.

**Table 4:** Discriminant validity—Cross-loadings.

	Men									Women									Total								
	BFS			AFS	FSB					BFS			AFS	FSB					BFS			AFS	FSB				
	EC	CT	FC		PH	EH	FH	CC	SC	EC	CT	FC		PH	EH	FH	CC	SC	EC	CT	FC		PH	EH	FH	CC	SC
BFS1	0.807	0.296	0.190	0.104	0.184	0.186	0.114	0.168	0.160	0.879	0.123	0.254	0.092	0.209	0.119	0.166	0.141	0.097	0.846	0.202	0.221	0.096	0.203	0.156	0.147	0.160	0.126
BFS2	0.888	0.298	0.322	0.164	0.222	0.199	0.163	0.214	0.217	0.852	0.183	0.303	0.130	0.182	0.106	0.168	0.065	0.041	0.873	0.235	0.300	0.147	0.214	0.156	0.175	0.151	0.121
BFS3	0.326	0.938	0.475	0.222	0.245	0.306	0.171	0.288	0.223	0.199	0.861	0.444	0.016	0.119	0.234	0.094	0.087	0.139	0.255	0.910	0.458	0.114	0.177	0.269	0.132	0.182	0.177
BFS4	0.304	0.865	0.481	0.171	0.144	0.254	0.003	0.216	0.136	0.110	0.886	0.487	0.080	0.157	0.259	0.079	0.086	0.110	0.192	0.866	0.484	0.116	0.143	0.256	0.041	0.144	0.123
BFS5	0.211	0.232	0.647	0.180	0.207	0.264	0.270	0.220	0.223	0.293	0.184	0.659	0.255	0.275	0.255	0.261	0.233	0.138	0.256	0.207	0.655	0.220	0.238	0.256	0.264	0.224	0.173
BFS6	0.301	0.390	0.762	0.255	0.190	0.347	0.239	0.262	0.244	0.226	0.429	0.777	0.121	0.285	0.336	0.175	0.180	0.171	0.246	0.408	0.772	0.180	0.230	0.336	0.201	0.211	0.202
BFS7	0.236	0.464	0.786	0.249	0.215	0.344	0.180	0.320	0.287	0.205	0.484	0.797	0.146	0.245	0.304	0.199	0.125	0.164	0.210	0.478	0.793	0.189	0.221	0.316	0.185	0.208	0.217
BFS8	0.162	0.456	0.759	0.226	0.240	0.330	0.128	0.220	0.242	0.220	0.536	0.764	0.138	0.279	0.238	0.207	0.129	0.114	0.192	0.492	0.762	0.169	0.253	0.275	0.168	0.164	0.168
AFS6	0.167	0.254	0.272	0.757	0.269	0.348	0.314	0.370	0.364	0.022	0.110	0.130	0.635	0.157	0.260	0.261	0.338	0.384	0.080	0.176	0.188	0.688	0.202	0.295	0.279	0.345	0.374
AFS7	−0.007	0.172	0.200	0.720	0.255	0.277	0.385	0.323	0.327	0.037	0.088	0.139	0.655	0.189	0.239	0.312	0.284	0.351	0.018	0.124	0.164	0.688	0.221	0.257	0.345	0.302	0.339
AFS8	0.106	0.067	0.186	0.736	0.237	0.210	0.442	0.317	0.336	0.161	0.040	0.193	0.787	0.299	0.265	0.510	0.297	0.303	0.143	0.048	0.187	0.765	0.272	0.239	0.475	0.310	0.317
AFS9	0.077	0.058	0.155	0.675	0.245	0.189	0.354	0.199	0.274	0.160	−0.064	0.116	0.771	0.244	0.159	0.355	0.287	0.182	0.132	−0.013	0.129	0.651	0.246	0.175	0.354	0.250	0.222
AFS10	0.179	0.125	0.267	0.681	0.164	0.216	0.283	0.263	0.310	0.184	−0.011	0.140	0.693	0.292	0.209	0.293	0.227	0.253	0.188	0.049	0.188	0.686	0.232	0.214	0.289	0.250	0.279
FSB1	0.128	0.018	0.145	0.208	0.743	0.499	0.498	0.410	0.448	0.171	0.034	0.248	0.283	0.820	0.453	0.507	0.449	0.341	0.158	0.021	0.195	0.249	0.789	0.477	0.505	0.433	0.390
FSB2	0.222	0.218	0.234	0.355	0.841	0.587	0.519	0.424	0.433	0.197	0.145	0.328	0.226	0.828	0.505	0.472	0.392	0.361	0.216	0.179	0.272	0.293	0.831	0.549	0.499	0.414	0.394
FSB3	0.185	0.166	0.217	0.260	0.815	0.563	0.485	0.483	0.433	0.247	0.114	0.305	0.302	0.871	0.532	0.534	0.423	0.325	0.228	0.134	0.257	0.281	0.848	0.550	0.513	0.457	0.374
FSB4	0.215	0.255	0.294	0.275	0.803	0.580	0.403	0.421	0.390	0.114	0.221	0.298	0.287	0.727	0.564	0.490	0.457	0.418	0.175	0.230	0.287	0.281	0.768	0.574	0.447	0.445	0.401
FSB5	0.182	0.320	0.245	0.158	0.462	0.665	0.278	0.281	0.241	0.040	0.285	0.304	0.197	0.366	0.700	0.331	0.287	0.304	0.108	0.302	0.275	0.178	0.411	0.681	0.308	0.283	0.276
FSB6	0.172	0.358	0.380	0.310	0.531	0.786	0.362	0.452	0.382	0.090	0.263	0.295	0.251	0.457	0.768	0.388	0.386	0.436	0.127	0.308	0.330	0.279	0.492	0.778	0.375	0.416	0.411
FSB7	0.216	0.140	0.368	0.338	0.604	0.825	0.583	0.568	0.543	0.146	0.140	0.274	0.299	0.598	0.751	0.489	0.461	0.356	0.187	0.134	0.312	0.316	0.605	0.786	0.538	0.516	0.441
FSB8	0.107	0.128	0.288	0.272	0.489	0.713	0.486	0.448	0.490	0.099	0.085	0.190	0.249	0.355	0.600	0.390	0.390	0.379	0.099	0.103	0.232	0.257	0.419	0.657	0.433	0.417	0.429
FSB9	0.125	0.139	0.227	0.326	0.392	0.451	0.709	0.449	0.379	0.136	−0.010	0.169	0.441	0.457	0.436	0.763	0.390	0.395	0.136	0.059	0.193	0.384	0.426	0.447	0.739	0.422	0.389
FSB10	0.091	−0.078	0.099	0.373	0.285	0.301	0.744	0.410	0.407	0.144	−0.017	0.108	0.429	0.338	0.293	0.760	0.336	0.351	0.128	−0.049	0.102	0.401	0.319	0.297	0.752	0.377	0.376
FSB11	0.13	−0.001	0.130	0.340	0.499	0.441	0.744	0.479	0.509	0.224	0.004	0.211	0.361	0.601	0.412	0.756	0.515	0.365	0.187	−0.002	0.172	0.351	0.555	0.427	0.753	0.499	0.430
FSB12	0.143	0.116	0.291	0.367	0.539	0.446	0.730	0.501	0.485	0.217	0.084	0.199	0.306	0.541	0.419	0.719	0.449	0.353	0.187	0.095	0.239	0.335	0.541	0.431	0.725	0.474	0.413
FSB13	0.113	0.214	0.256	0.323	0.432	0.438	0.625	0.434	0.484	0.079	0.245	0.331	0.340	0.389	0.496	0.636	0.412	0.473	0.098	0.227	0.294	0.333	0.413	0.467	0.636	0.423	0.477
FSB15	0.109	0.108	0.176	0.421	0.369	0.393	0.713	0.504	0.467	0.024	0.155	0.227	0.336	0.339	0.378	0.672	0.379	0.444	0.069	0.127	0.200	0.381	0.357	0.383	0.690	0.443	0.454
FSB16	0.209	0.225	0.257	0.348	0.369	0.450	0.466	0.824	0.572	0.067	0.158	0.190	0.321	0.365	0.450	0.441	0.806	0.575	0.137	0.189	0.215	0.336	0.372	0.450	0.457	0.818	0.573
FSB17	0.182	0.259	0.332	0.410	0.538	0.558	0.633	0.880	0.578	0.131	0.012	0.185	0.353	0.500	0.433	0.500	0.842	0.476	0.166	0.125	0.242	0.381	0.527	0.495	0.566	0.860	0.520
FSB18	0.165	0.146	0.271	0.435	0.536	0.482	0.617	0.603	0.768	0.078	0.128	0.184	0.378	0.382	0.435	0.539	0.562	0.801	0.108	0.136	0.222	0.404	0.448	0.453	0.570	0.571	0.789
FSB19	0.18	0.234	0.290	0.284	0.343	0.484	0.434	0.473	0.741	0.053	0.108	0.141	0.350	0.346	0.416	0.389	0.467	0.748	0.112	0.680	0.198	0.321	0.347	0.446	0.410	0.470	0.742
FSB20	0.122	0.145	0.197	0.356	0.311	0.357	0.398	0.437	0.763	0.019	0.130	0.149	0.270	0.261	0.362	0.329	0.411	0.690	0.064	0.133	0.169	0.310	0.285	0.358	0.359	0.422	0.725
FSB21	0.222	0.130	0.283	0.406	0.401	0.411	0.496	0.545	0.819	0.090	0.073	0.130	0.370	0.352	0.370	0.401	0.490	0.811	0.150	0.096	0.192	0.388	0.379	0.386	0.444	0.515	0.814

Note. BFS = Background Food Safety; AFS = Attitudes Food Safety; FSB = Food Safety Behavior; EC = Cooking Experience; CT = Courses Taken; FC = Source of Knowledge; PH = Personal Hygiene; EH = Equipment Hygiene; FH = Food Hygiene; CC = Cross-Contamination; SC = Storage Conditions.

**Table 5:** Discriminant validity using the Fornell-Larcker criterion and the Heterotrait-Monotrait method (HTMT).

	BFS	AFS	FSB	EC	CT	FC	PH	EH	FH	CC	SC
Men											
BFS	*0.771*	0.39	0.534				0.42	0.597	0.353	0.575	0.463
AFS	0.299	*0.715*	0.599	0.227	0.248	0.407	0.406	0.447	0.63	0.592	0.574
FSB	0.422	0.5	*0.827*	0.353	0.319	0.509					
EC		0.15	0.262	*0.848*	0.504	0.449	0.326	0.334	0.233	0.364	0.317
CT		0.201	0.275	0.349	*0.903*	0.699	0.258	0.41	0.192	0.397	0.261
FC		0.307	0.409	0.31	0.525	*0.74*	0.363	0.582	0.376	0.509	0.447
HP	0.327	0.328		0.241	0.224	0.287	*0.802*	0.885	0.732	0.745	0.651
HE	0.44	0.356		0.228	0.313	0.435	0.699	*0.75*	0.742	0.849	0.727
HA	0.252	0.494		0.166	0.11	0.275	0.588	0.575	*0.712*	0.906	0.795
CC	0.38	0.421		0.227	0.285	0.348	0.54	0.596	0.652	*0.852*	0.953
CA	0.344	0.455		0.225	0.207	0.338	0.526	0.562	0.64	0.674	*0.773*
Women											
BFS	*0.748*	0.299	0.454				0.468	0.545	0.379	0.353	0.273
AFS	0.206	*0.711*	0.626	0.185	0.165	0.303	0.419	0.49	0.64	0.639	0.584
FSB	0.353	0.497	*0.805*	0.245	0.251	0.447					
EC		0.162	0.192	*0.866*	0.264	0.451	0.301	0.197	0.263	0.197	0.108
CT		0.049	0.189	0.175	*0.874*	0.759	0.209	0.403	0.164	0.17	0.198
FC		0.208	0.361	0.321	0.532	*0.751*	0.461	0.531	0.37	0.356	0.26
HP	0.359	0.336		0.227	0.157	0.364	*0.813*	0.848	0.754	0.796	0.554
HE	0.369	0.324		0.131	0.281	0.38	0.633	*0.708*	0.772	0.911	0.731
HA	0.28	0.501		0.199	0.094	0.283	0.623	0.562	*0.719*	0.876	0.692
CC	0.218	0.404		0.121	0.099	0.229	0.527	0.536	0.575	*0.824*	0.996
CA	0.195	0.419		0.082	0.141	0.197	0.442	0.517	0.545	0.635	*0.765*
Total Sample											
BFS	*0.758*	0.327	0.492				0.443	0.571	0.368	0.465	0.359
AFS	0.239	*0.712*	0.601	0.223	0.161	0.321	0.417	0.45	0.625	0.615	0.557
FSB	0.379	0.497	*0.816*	0.306	0.281	0.463					
EC		0.156	0.229	*0.86*	0.364	0.437	0.328	0.265	0.258	0.294	0.2
CT		0.113	0.225	0.255	*0.888*	0.728	0.221	0.405	0.173	0.284	0.227
FC		0.244	0.371	0.306	0.527	*0.747*	0.402	0.547	0.36	0.414	0.337
HP	0.336	0.33		0.242	0.18	0.316	*0.81*	0.865	0.745	0.773	0.596
HE	0.4	0.337		0.182	0.294	0.398	0.668	*0.728*	0.756	0.875	0.726
HA	0.266	0.494		0.191	0.099	0.276	0.605	0.568	*0.717*	0.89	0.738
CC	0.29	0.413		0.181	0.185	0.273	0.54	0.565	0.613	*0.839*	0.964
CA	0.261	0.436		0.144	0.171	0.256	0.481	0.536	0.587	0.649	*0.769*

Note. The italicized diagonal elements correspond to the square roots of the AVE (average variance extracted). The values below the diagonal represent the correlations between constructs, whereas the values above the diagonal correspond to the HTMT indices. BFS = Background Food Safety; AFS = Attitudes Food Safety; FSB = Food Safety Behavior; EC = Cooking Experience; CT = Courses Taken; FC = Source of Knowledge; PH = Personal Hygiene; EH = Equipment Hygiene; FH = Food Hygiene; CC = Cross-Contamination; SC = Storage Conditions.

**Table 6:** Direct relationships (Hypotheses H1 to H3).

	Men			Women			Total Sample		
	β	T	*p*	β	T	*p*	β	T	*p*
**H1: BFS → FSB**	0.286	7.143	0	0.261	6.526	0	0.266	9.223	0
**H2: AFS → FSB**	0.445	9.562	0	0.472	11.42	0	0.457	14.2	0
**H3: BFS → AFS**	0.319	5.853	0	0.209	3.416	0	0.249	6.505	0

Note. BFS = Background in Food Safety; AFS = Attitudes in Food Safety; FSB = Food Safety Behavior; β: Structural paths, T: Student’s *t*-test, *p*: *p*-value.

**Table 7:** Mediation analysis (Hypothesis 4).

	Men			Women			Total Sample		
	β	T	*p*	β	T	*p*	β	T	*p*
**H4: BFS → AFS → FSB**	0.141	5.293	0.000	0.100	3.181	0.000	0.114	5.692	0.000

Note. β = path coefficient; T = Student’s t statistic; *p* = *p*-value; BFS = Background Food Safety; AFS = Attitudes Food Safety; FSB = Food Safety Behavior.

**Table 8:** Multigroup comparison (Hypothesis 5).

Path	Path Difference (Men-Women)	*p*-Value (Men-Women)
**H5 a: BFS → FSB**	0.027	0.303
**H5 b: BFS → AFS**	0.106	0.088
**H5 c: AFS → FSB**	0.028	0.672

Note. Path difference = difference in standardized path coefficients between men and women. BFS = Background Food Safety; AFS = Attitudes Food Safety; FSB = Food Safety Behavior.

## Data Availability

The original contributions presented in this study are included in the article. Further inquiries can be directed to the corresponding author.

## Appendix A. Food Safety Background, Attitudes, and Behavior

### Appendix A.1. Food Safety Background Factors

Response scale for items 1–4/Escala de respuesta para los ítems 1–4

1 = Never, 2 = Almost never, 3 = Sometimes, 4 = Almost every day, 5 = Every day;

1 = Nunca, 2 = Casi nunca, 3 = En ocasiones, 4 = Casi todos los días, 5 = Todos los días

**English Version**	**Versión En Español**
Experience in cooking and food handling	Experiencia en cocina y manipulación de alimentos
1. How often in the past year have you cooked for yourself or for somebody else in your household: food from raw ingredients such as minced meat, fish, or chicken?	1. Durante el último año, ¿con qué frecuencia manipulaste carne molida, pescado o pollo?
2. How often in the past year have you cooked for yourself or for somebody else in your household: fresh vegetables/root vegetables/leeks/potatoes?	2. Durante el último año, ¿con qué frecuencia manipulaste verduras, tubérculos, poros o frutas?
Food safety courses completed	Cursos tomados en inocuidad alimentaria
3. How often have you taken courses in food hygiene/safety and/or microbiology at high school?	3. ¿Con qué frecuencia ha tomado cursos sobre higiene/inocuidad alimentaria y/o microbiología en el colegio secundario?
4. How often have you taken courses in food hygiene/safety and/or microbiology at university/college?	4. ¿Con qué frecuencia ha tomado cursos sobre higiene/inocuidad alimentaria y/o microbiología en la universidad?

Response scale for items 5–8/Escala de respuesta para los ítems 5–8

1 = Strongly disagree, 2 = Disagree, 3 = Neither agree nor disagree, 4 = Agree, 5 = Strongly agree

1 = Totalmente en desacuerdo, 2 = En desacuerdo, 3 = Ni de acuerdo ni en desacuerdo, 4 = De acuerdo, 5 = Totalmente de acuerdo

**English Version**	**Versión En Español**
Source of food safety knowledge	Fuente de conocimiento en inocuidad alimentaria
5. My foremost food safety source of knowledge is my mother/female relative.	5. Mi madre u otro miembro de mi familia me enseñó mucho sobre cómo mantener los alimentos inocuos y libres de riesgos.
6. My foremost food safety source of knowledge is my partner/friend.	6. Aprendí mucho sobre cómo mantener los alimentos inocuos a través de mi pareja o amigo(a).
7. My foremost food safety source of knowledge is a course taken at high school.	7. Aprendí mucho sobre inocuidad alimentaria en algún curso que estudié en el colegio secundario.
8. My foremost food safety source of knowledge is a course taken at university/college.	8. Aprendí mucho sobre inocuidad alimentaria en algún curso que estudié en la universidad.

### Appendix A.2. Food Safety Attitudes

Response scale/Escala de respuesta

1 = Strongly disagree, 2 = Disagree, 3 = Neither agree nor disagree, 4 = Agree, 5 = Strongly agree

1 = Totalmente en desacuerdo, 2 = En desacuerdo, 3 = Ni de acuerdo ni en desacuerdo, 4 = De acuerdo, 5 = Totalmente de acuerdo

**English Version**	**Versión En Español**
1. The conditions (cleanliness, hygiene, dampness, etc.) of the places where the food is sold are very important.	1. Las condiciones (limpieza, higiene, humedad, etc.) del lugar donde se venden los alimentos son muy importantes.
2. Dirty eggs can be broken and used carefully without washing.	2. Los huevos de gallina sucios pueden romperse y usarse sin lavar, pero con cuidado.
3. The appearance of the food is more important than hygiene.	3. La apariencia de los alimentos es más importante que la higiene.
4. Consuming moldy cheese is beneficial for health.	4. Consumir queso con moho es bueno para la salud.
5. There is nothing wrong in using sprouted potatoes and onions.	5. No tiene nada de malo usar cebollas y papas germinadas.
6. After touching raw foods, cooked foods should not be touched.	6. No se debe tocar alimentos cocidos después de tocar alimentos crudos.
7. Meatballs, pastries, etc., can be prepared with bare hands.	7. Las albóndigas, pasteles, etc., se pueden preparar con las manos descubiertas.
8. Safe food is not directly related to human health.	8. La seguridad de los alimentos no está directamente relacionada con la salud del ser humano.
9. Additional information on food safety needs to be learned.	9. Se necesita aprender más sobre seguridad alimentaria.
10. Foodborne diseases are common.	10. Las enfermedades transmitidas por alimentos son frecuentes.
11. It is safe to consume foods prepared outside the home.	11. Es seguro consumir alimentos preparados fuera del hogar.

### Appendix A.3. Food Safety Behavior

Response scale/Escala de respuesta

1 = Never, 2 = Almost never, 3 = Sometimes, 4 = Almost always, 5 = Always

1 = Nunca, 2 = Casi nunca, 3 = En ocasiones, 4 = Casi siempre, 5 = Siempre

**Table A1:** Personal Hygiene/Higiene personal.

English Version	Versión En Español
1. I wash my hands before eating or preparing food.	1. Me lavo las manos antes de comer o cocinar.
2. I use paper towels or handkerchiefs to dry my hands.	2. Uso un pañuelo o papel toalla para secar mis manos lavadas.
3. I wash my hands before eating and after.	3. Me lavo las manos antes y después de comer.
4. I take care to rub my hands with soap bubbles for at least 20 s while washing.	4. Me aseguro de frotar con la espuma del jabón durante 20 segundos como mínimo al lavarme las manos.

**Table A2:** Equipment Hygiene/Higiene de los equipos.

English Version	Versión En español
5. I chop the vegetables or fruits on the board that I used for meat.	5. Pico las frutas o verduras en la misma tabla que uso para la carne.
6. I wash or wipe cans, plastic, and glass containers before opening them.	6. Lavo o limpio las latas, envases de plástico y de vidrio antes de abrirlos.
7. Before preparing the food, I clean the surfaces I will use.	7. Antes de preparar la comida, limpio las superficies que utilizaré.
8. I leave the dishcloths I used in the kitchen in a way that they can dry.	8. Coloco los trapos de cocina usados de manera que puedan secarse.

**Table A3:** Food Hygiene/Higiene alimentaria.

English Version	Versión En Español
9. I use products like rice, lentils, and bulgur without washing.	9. Uso productos sin lavar como arroz, lentejas y trigo.
10. I cut the moldy parts of the moldy foods and consume the rest.	10. Quito las partes con moho de los alimentos y consumo el resto.
11. I consume vegetables and fruits after they are washed.	11. Lavo las frutas y verduras antes de consumirlas.
12. I check the expiry dates on food packages.	12. Reviso las fechas de vencimiento de los envases de alimentos.
13. I freeze the remainder of the defrosted products.	13. Si sobra parte de un producto descongelado, lo vuelvo a congelar.
14. I use raw milk instead of pasteurized milk.	14. Uso leche cruda en vez de leche pasteurizada.
15. I use broken or cracked eggs.	15. Uso huevos incluso si están rotos o agrietados.

**Table A4:** Cross-Contamination/Contaminación cruzada.

English Version	Versión En Español
16. I store raw foods and ready-to-eat foods in the refrigerator closed off and on separate shelves.	16. Guardo los alimentos crudos y cocinados en el refrigerador en estantes separados y cerrados.
17. After chopping raw meat, I wash my hands before touching another food.	17. Después de cortar la carne cruda, me lavo las manos antes de tocar otro alimento.

**Table A5:** Storage Conditions/Condiciones de almacenamiento.

English Version	Versión En Español
18. I keep dairy products, eggs, and meat products refrigerated at all times until use.	18. Mantengo los lácteos, huevos y productos cárnicos refrigerados en todo momento hasta su uso.
19. Meat, poultry, fish, and frozen foods are the last items I purchase when shopping.	19. La carne, el pollo, el pescado y los alimentos congelados son los últimos productos que adquiero al ir de compras.
20. I reheat prepared food only once after removing it from the refrigerator.	20. Caliento la comida preparada solo una vez después de sacarla del refrigerador.
21. I place foods (frozen items, meats, etc.) that can spoil quickly in the refrigerator within the first two hours after purchase.	21. Coloco los alimentos (congelados, carnes, etc.) que pueden malograrse en poco tiempo en el refrigerador dentro de las primeras dos horas después de la compra.
